# Near-Field Radiative Heat Transfer Between Heavily Phosphorus-Doped Silicon Plates: Effects of Doping Concentration

**DOI:** 10.3390/mi17080954

**Published:** 2026-08-12

**Authors:** Jincheng Wang, Ning Guo, Ronghui Yang, Kui Wang, Bosen Chen, Weiwei Tang

**Affiliations:** 1 School of Physics and Electronics, Hunan Normal University, Changsha 410081, China; wangjincheng@hunnu.edu.cn (J.W.); guoning@hunnu.edu.cn (N.G.); ronghuiyang@hunnu.edu.cn (R.Y.); 2 National Engineering and Technology Research Center for Emergency and Disaster Relief Equipment, PLA Joint Logistics Support Force University of Engineering, Chongqing 400042, China; kuiwang.nudt@hotmail.com; 3 Chongqing Gangli Environmental Protection Co., Ltd., Chongqing 400010, China

**Keywords:** near-field radiative heat transfer, heavily doped silicon, phosphorus doping, Drude model, surface plasmon polaritons

## Abstract

To address the critical thermal challenges in high-performance computing and three-dimensional integrated circuits, the doping-tunable control of near-field thermal radiation using CMOS-compatible materials offers a highly promising non-contact cooling strategy. In this work, radiative heat transfer between two parallel heavily phosphorus-doped silicon plates separated by a vacuum gap is studied using fluctuational electrodynamics. A doping-dependent Drude model is employed to describe the dielectric response of doped silicon, including carrier concentration, ionization, and mobility effects. The influences of gap width and doping concentration on the total and spectral heat transfer are systematically analyzed. The results show that the heat transfer increases sharply as the gap decreases and is mainly governed by TM-polarized evanescent modes. Under symmetric doping, the spectral peak shifts to higher frequencies as the doping concentration increases from $10^{18}$ to $10^{21}\;{\mathrm{cm}}^{-3}$, while the strongest transfer occurs at $10^{19}\;{\mathrm{cm}}^{-3}$ because of favorable surface-plasmon-polariton coupling and impedance matching. These findings provide a theoretical foundation for chip-scale thermal management and on-chip radiative cooling in CMOS-compatible silicon platforms, although practical implementation would require dynamic tuning mechanisms and device-level engineering in future work.

## 1. Introduction

When the separation distance between two bodies becomes smaller than the characteristic thermal wavelength, radiative heat transfer can exceed the blackbody limit by several orders of magnitude owing to evanescent-wave tunneling and surface-mode coupling. This physical framework was established by Polder and Van Hove [1] and has since served as the theoretical foundation of near-field radiative heat transfer. Because of this super-Planckian enhancement, near-field thermal radiation has attracted sustained interest in a broad range of applications, including thermal diodes [2], thermophotovoltaic systems [3,4], and solid-state refrigeration cycles [5], electroluminescent or radiation-assisted cooling concepts, hot-spot-sensitive micro- and nanoscale thermal technologies, and heat-assisted magnetic recording [6,7]. More broadly, recent reviews and perspective articles have shown that near-field thermal radiation is evolving from a fundamental topic in nanoscale heat transfer into a technologically relevant platform for energy conversion and thermal management [8]. Unlike far-field radiation, near-field radiative transfer depends not only on the temperature difference but also, more strongly, on the dielectric response of the interacting materials and the electromagnetic surface modes supported at their interfaces. Therefore, doping-tunable regulation of near-field thermal radiation requires material systems whose infrared optical properties can be tuned in a practical and robust manner.

In recent years, substantial progress has been made in manipulating near-field thermal radiation through material design, mode engineering, and many-body or structured configurations. On the theoretical side, the transition from classical fluctuational electrodynamics to application-oriented mode tailoring has been summarized in recent reviews [8] and further explored in plasmonic and structured radiative systems [9,10]. For nanoscale energy conversion and transport, the roles of spectral selectivity, resonant photon tunneling, and material compatibility have been highlighted in recent studies [11,12,13,14]. Related advances in mode-enhanced or anisotropy-assisted near-field heat transfer have also been reported in polaritonic and low-dimensional material systems [15,16]. In parallel, recent studies have further demonstrated that spectral heat flux, transmission channels, and interfacial coupling can be effectively manipulated by tailoring optical dispersion and surface-wave behavior [17,18,19,20]. These developments indicate that the central issue is no longer merely how to enhance near-field heat transfer, but how to realize thermal radiation platforms that are dynamically tunable, physically transparent, and compatible with practical fabrication. This issue is particularly important for chip-scale thermal management. With the rapid development of high-performance computing and three-dimensional integrated circuits, continuously increasing power density and localized hot spots have become major challenges to device reliability and operational stability. Under such conditions, non-contact thermal regulation based on radiative transfer offers an attractive complement to conventional conduction-dominated cooling strategies. However, many high-performance near-field platforms rely on polar materials, heterostructures, metamaterials, twisted low-dimensional systems, or nanostructures with considerable fabrication complexity [11,12,13,14,15,16,17,18,19]. Although these platforms are highly valuable for revealing fundamental mechanisms, their direct integration into standard semiconductor processing routes remains nontrivial. Consequently, CMOS-compatible materials whose infrared dielectric response can be tuned intrinsically, rather than solely through complex structural patterning, are of particular interest for practical on-chip thermal control.

Heavily phosphorus-doped silicon is a representative candidate in this context. As a technologically mature and CMOS-compatible material, doped silicon provides a highly tunable free-carrier concentration and therefore a controllable infrared dielectric response. The infrared optical and radiative properties of heavily doped silicon have been systematically studied by Basu, Lee, and Zhang [21,22], who showed that a doping-dependent Drude description can capture the essential contribution of free carriers. Earlier, Fu and Zhang [23] demonstrated that near-field radiative heat transfer between silicon media depends strongly on the doping level and that spectral transport can be significantly altered by variations in carrier concentration. In the heavily n-doped regime, silicon can exhibit plasmonic behavior in the infrared and support surface plasmon polaritons, thereby providing efficient channels for resonant photon tunneling across nanometric vacuum gaps. Compared with heterogeneous plasmonic materials, doped silicon offers a materially simple and technologically relevant platform in which radiative behavior can be controlled primarily through carrier concentration. This feature is also consistent with the broader trend toward CMOS-compatible infrared plasmonics [17]. Despite these advances, several issues remain insufficiently understood for systems composed of heavily phosphorus-doped silicon plates. Existing studies have established that the dielectric function of doped silicon is strongly influenced by carrier density, ionization, and mobility, with impurity ionization described by Kuzmicz [24] and related transport-property modeling forming the basis for subsequent radiative analyses [21,22,23]. Nevertheless, a systematic understanding is still lacking of how phosphorus doping concentration governs the evolution of dielectric response, the redistribution of spectral heat flux, and the characteristics of transmission channels in two parallel heavily doped silicon plates, especially under symmetric and asymmetric doping configurations relevant to dynamic thermal regulation. In particular, it remains necessary to clarify why increasing doping does not always lead to stronger near-field heat transfer, how the spectral peaks shift and broaden with doping concentration, and under what conditions coupled surface plasmon polaritons produce optimal photon tunneling and maximum heat-transfer performance.

Motivated by these considerations, this work theoretically investigates near-field radiative heat transfer between two parallel heavily phosphorus-doped silicon plates separated by a vacuum gap. The analysis is conducted within the framework of fluctuational electrodynamics originally formulated by Polder and Van Hove [1], while the dielectric response of doped silicon is described by a doping-dependent Drude model following the physical treatment established in previous studies on heavily doped silicon [21,22,23], with carrier ionization and mobility effects incorporated consistently [24]. We first examine the dielectric properties of heavily doped silicon and then systematically analyze the effects of vacuum-gap distance, asymmetric doping, and symmetric doping on the total heat transfer, spectral heat flux, and energy transmission coefficient. Particular attention is paid to the physical origin of the optimal heat-transfer condition and to the role of coupled surface plasmon polaritons in the two-plate system. By clarifying the interplay among doping-dependent optical response, mode coupling, and photon tunneling, this study aims to provide a clearer physical basis for CMOS-compatible near-field thermal regulation by elucidating the fundamental mechanisms of doping-tunable radiative transfer, thereby offering useful theoretical guidance for chip-scale thermal management and hot-spot mitigation. It should be noted that dynamic tuning mechanisms and device-level analyses are not addressed here and are left for future investigations.

## 2. Theoretical Model and Method

### 2.1. Physical Configuration

**Figure 1:**
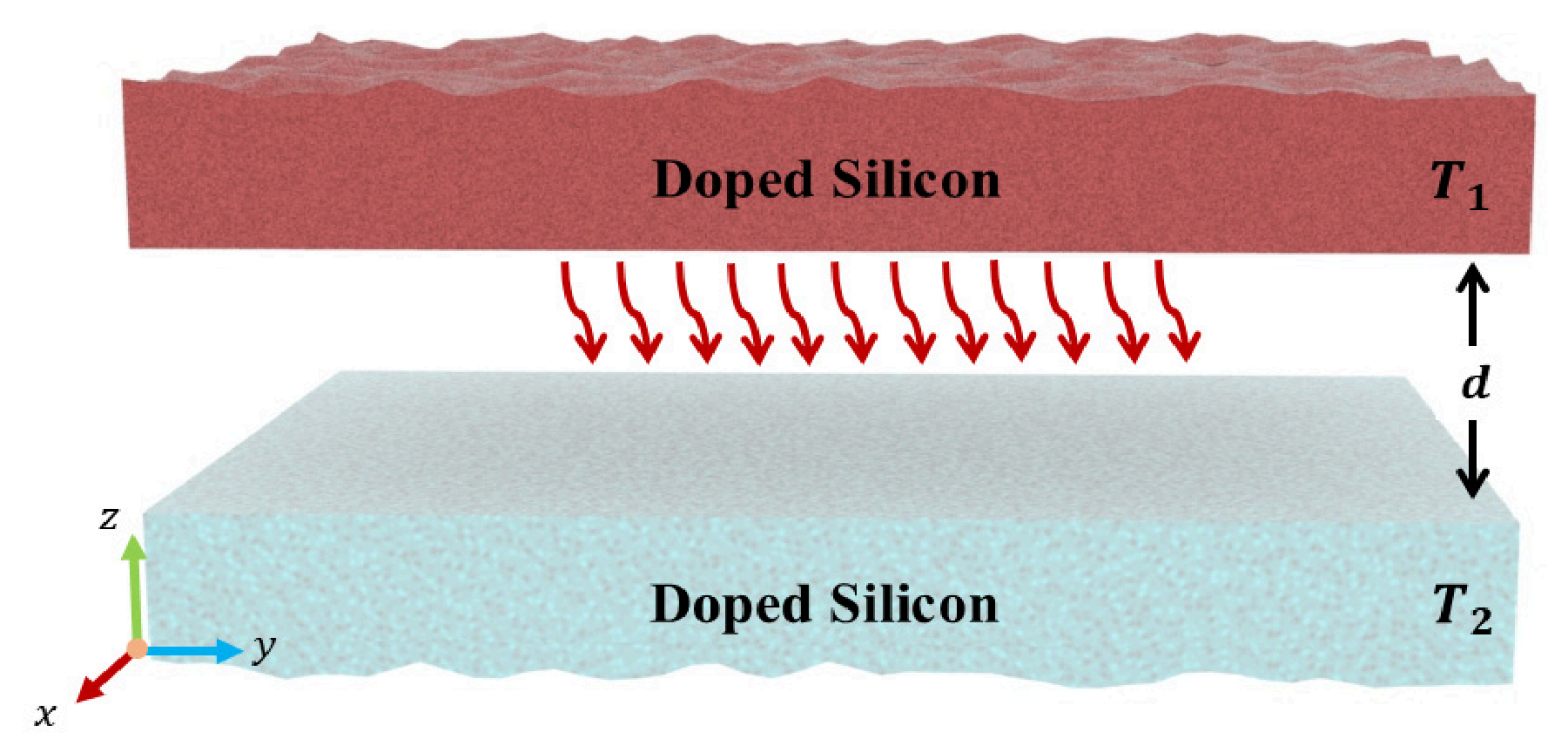
Schematic of near-field radiative heat transfer between two semi-infinite heavily phosphorus-doped silicon (n-type D-Si) plates separated by a vacuum gap d.

The system considered in this work is shown schematically in Figure 1. It consists of two semi-infinite heavily phosphorus-doped silicon plates separated by a vacuum gap of width d. Hereafter, heavily phosphorus-doped silicon is denoted as n-type D-Si. The upper plate is maintained at $T_{1}=400\;K$, while the lower plate is held at $T_{2}=300\;K$, to establish a nonequilibrium thermal condition. Unless otherwise specified, the initial vacuum gap is set to d=10 nm. This planar geometry provides a standard configuration for studying photon tunneling and near-field thermal radiation. At nanoscale separations, thermally excited evanescent fields generated at one interface can tunnel through the vacuum gap and couple to the modes of the opposite interface, leading to a strong enhancement in radiative heat transfer.

It should be noted that the present model assumes ideal planar interfaces with atomic-scale smoothness, infinite lateral dimensions, and a perfectly uniform vacuum gap. In realistic CMOS-compatible devices, surface roughness, finite-size effects, fabrication-induced defects, and gap nonuniformity may introduce additional scattering channels and reduce the spectral coherence of surface plasmon polaritons, thereby weakening the predicted near-field enhancement to some extent. Nevertheless, the primary objective of this work is to elucidate the intrinsic physical mechanisms governing doping-tunable near-field radiative transfer, particularly the role of symmetric versus asymmetric doping configurations and the associated SPP coupling conditions, rather than to account for engineering-specific corrections. A systematic investigation of these practical effects is not yet available in the literature for heavily doped silicon systems, and we explicitly identify this as a natural and important direction for future research. The present theoretical framework is intended to provide a solid foundation for such subsequent extensions toward practical device applications.

### 2.2. Near-Field Radiative Heat Transfer Formulation

Within the framework of fluctuational electrodynamics, the net radiative heat flux can be written as follows:

(1) $$H(T_{1},T_{2})=\int_{0}^{\infty }\frac{d\omega }{{\left(2\pi \right)}^{2}}[\Theta (\omega ,T_{1})-\Theta (\omega ,T_{2})]\int_{0}^{\infty }\xi (\omega ,\beta )\beta d\beta ,$$

where $\beta =\sqrt{k_{x}^{2}+k_{y}^{2}}$ is the in-plane wave vector, and $\Theta (\omega ,T)=\hbar \omega /\exp[(\hbar \omega /k_{B}T)-1]$ is a function related to the Planck oscillator’s average energy as a function of the angular frequency ω at a given temperature T. The energy transfer coefficient contribution of the propagation mode and the evanescent mode is represented by $\xi (\omega ,\beta )=\xi^{s}(\omega ,\beta )+\xi^{p}(\omega ,\beta )$, and this can be written as [25]:

(2) ξj(ω,β)=(1−r1j2)(1−r2j2)1−r1jr2je2ikzd2,β<ω/c4Im{r1j}Im{r2j}e−2kzd1−r1jr2je2ikzd2,β>ω/c,

where $r_{i}^{j}(j=s,p;i=1,2)$ are the reflection coefficients for TM (p-polarized) and TE (s-polarized) modes in the dielectric slab i. Under the semi-infinite plate model, the transmission coefficients are negligible. For vacuum incidence, the Fresnel coefficients are expressed as:

(3) $$r_{i,s}=\frac{k_{z}-k_{i,z}}{k_{z}+k_{i,z}},$$

(4) $$r_{i,P}=\frac{\varepsilon_{i}k_{z}-k_{i,z}}{\varepsilon_{i}k_{z}+k_{i,z}},$$

where $k_{i,z}=\sqrt{\varepsilon_{i}k_{0}^{2}-\beta^{2}}$ is the out-of-plane wave vector for the dielectric plate i, and $\varepsilon_{i}$ is the corresponding dielectric constant of the plate, and can be expressed as:

(5) $$\varepsilon (\omega )=\varepsilon_{\infty }-\frac{{\omega_{p}}^{2}}{\omega^{2}+i\omega /\tau },$$

where the band gap transitions and contribution of lattice vibration are indicated by [26]. Plasma frequency and scattering time are represented by $\omega_{p}$ and τ respectively. Here, $\varepsilon_{\infty }$ is regarded as 11.7, and plasma frequency $\omega_{p}=\sqrt{N_{e}e^{2}/(m^{*}\varepsilon_{0})}$. Where e, $N_{e}$ indicate the electron charge and electron concentration, and $\varepsilon_{0}$, $m^{*}$ designate permittivity in a vacuum and the electron effective mass, respectively. It is assumed that $m^{*}$ is independent of doping concentration, frequency, and temperature, where it takes the $m^{*}$ as $0.27m_{e}$ and $m_{e}$ refers to the mass of a free electron in vacuum [20,26]. Whereas the τ of the free electron can be presented as [21]:

(6) $$\frac{1}{\tau }=\frac{1}{\tau_{e-d}}+\frac{1}{\tau_{e-l}},$$

where the scattering times of electrons in the lattice and of electrons with defects are $\tau_{e-l}$ and $\tau_{e-d}$ respectively. For high temperature conditions (400 K), it is necessary to consider both the impurity scattering and impurity lattice scattering. At this point, $\tau_{e-d}=m^{*}\mu_{e}/e$ and the electron mobility μ under temperature conditions (300 K) is given as in [20]:

(7) $$\mu =\mu_{1}+\frac{\mu_{\max}-\mu_{1}}{1+{\left(N_{e}/C_{r}\right)}^{\alpha }}-\frac{\mu_{2}}{1+{\left(C_{s}/N_{e}\right)}^{\lambda }},$$

here, $\mu_{1}=68.5\;cm^{2}/Vs$, $\mu_{\max}=1414\;cm^{2}/Vs$, $\mu_{2}=56.1\;cm^{2}/Vs$, $C_{s}=3.41\times 10^{20}\;cm^{-3}$, $C_{r}=9.2\times 10^{16}\;cm^{-3}$, α=0.711, λ=1.98. The degree of ionization ζ and doping concentration n decide the electron concentration $N_{e}=n\times \zeta$, and here the degree of ionization model is presented as:

(8) $$\zeta =1-A\;\exp\{-B\;\ln{\left(n/n_{0}\right)}^{2}\},$$

where the constants A, B, and $n_{0}$ are determined as follows. For n-type D-Si: $A=0.0824{\Theta_{T}}^{-1.622}$, $n_{0}=1.6\times 10^{18}{\Theta_{T}}^{0.7267}$, and $B=0.4722{\Theta_{T}}^{0.0652}$ for $n<n_{0}$, and $B=1.23-0.3162\Theta_{T}$ for $n\geq n_{0}$. Here, $\Theta_{T}=T/300$ is the normalized temperature, and T is expressed in Kelvin [20,25]. The electron mobility $\mu_{e}$ at a high temperature (400 K) is presented as [22]:

(9) $$\mu_{e}=\mu {\Theta_{T}}^{1.5},$$

where μ is the electron mobility under temperature conditions (300 K) as discussed above. For the lattice scattering, the scattering time $\tau_{e-l}$ at a high temperature (400 K) can be obtained by:

(10) $$\tau_{e-l}=\tau_{e-l}^{0}{\Theta_{T}}^{-3.8},$$

where $\tau_{e-l}^{0}=2.23\times 10^{-13}\;s$ [21]. The numerical integration was performed with a frequency cutoff of $5\times 10^{14}\;\mathrm{rad}/s$ and a grid of 500×11,000 points in the (ω,β) space. These parameters were chosen to ensure full convergence, with further grid refinement yielding relative changes of less than 0.06% in the spectral thermal conductance. The accuracy of the present computational implementation was further validated by comparing with the established results for doped silicon plates under identical parameters [25]. As a result, Figure 2 shows the dielectric functions’ real and imaginary parts (Re and Im) of n-type D-Si with different doping concentrations at 400 K. The computational accuracy has been carefully ensured by a fine frequency sampling step of 0.125%, and the influences of key Drude parameters are adopted from established literature [21,22], with a more detailed sensitivity analysis left for future investigation. The values of carrier concentration, mobility, and relaxation time at 300 K and 400 K for each doping concentration are listed in Table 1.

This model enables the dielectric response of heavily doped silicon to be evaluated as a function of doping concentration, ionization, and mobility, which is essential for analyzing the near-field radiative properties of the two-plate system.

## 3. Results and Discussion

### 3.1. Dielectric Response of Heavily Doped Silicon

To establish the material basis for the radiative transfer characteristics, we first examine the dielectric response of n-type D-Si at T=400 K. Figure 2 shows the real and imaginary parts of the dielectric function for doping concentrations ranging from $10^{18}$ to $10^{21}\;{\mathrm{cm}}^{-3}$. Moreover, the imaginary part of the dielectric function decreases monotonically with increasing angular frequency for all doping concentrations. By contrast, the real part increases with frequency, evolving from large negative values toward less negative values and eventually approaching positive values at sufficiently high frequencies. The free-carrier response is strongest for $n=10^{18}\;{\mathrm{cm}}^{-3}$, followed by $n=10^{19}\;{\mathrm{cm}}^{-3}$, as reflected by the large magnitudes of both Re and Im in the low-frequency region. For $n=10^{20}\;{\mathrm{cm}}^{-3}$ and $n=10^{21}\;{\mathrm{cm}}^{-3}$, the imaginary part remains comparatively small over the plotted range, and the real part varies more mildly.

These trends indicate that the dielectric response of heavily doped silicon can be effectively tuned by the doping concentration. Since the TM reflection coefficient and the SPP resonance condition depend directly on ε(ω), the behavior shown in Figure 2 is expected to strongly influence the spectral position, intensity, and bandwidth of near-field radiative heat transfer.

To place the present work in the context of previous theoretical investigations, Table 2 summarizes the key differences between this study and the most relevant prior reports on doped-silicon near-field radiative transfer. Compared with earlier works that focused primarily on the overall doping effect without systematically distinguishing symmetric and asymmetric configurations, the present study provides a comprehensive comparison of both doping schemes over a broad concentration range from $10^{18}$ to $10^{21}\;{\mathrm{cm}}^{-3}$. In addition, beyond calculating the total and spectral heat fluxes, we analyze the energy transmission coefficient in the (ω,β) plane to directly visualize the evolution of evanescent transmission channels with doping concentration. Quantitative analyses of SPP dispersion relations are also provided to support the physical interpretation of the optimal heat transfer condition at $n=10^{19}\;{\mathrm{cm}}^{-3}$. Furthermore, by focusing on phosphorus-doped silicon, a technologically mature and CMOS-compatible material, this study aims to offer more directly applicable guidance for chip-scale thermal management and on-chip radiative cooling. These distinct aspects are systematically presented in Table 2 and will be elaborated in detail in the following subsections.

### 3.2. Effect of Gap Distance on Total Radiative Heat Flux

**Figure 3:**
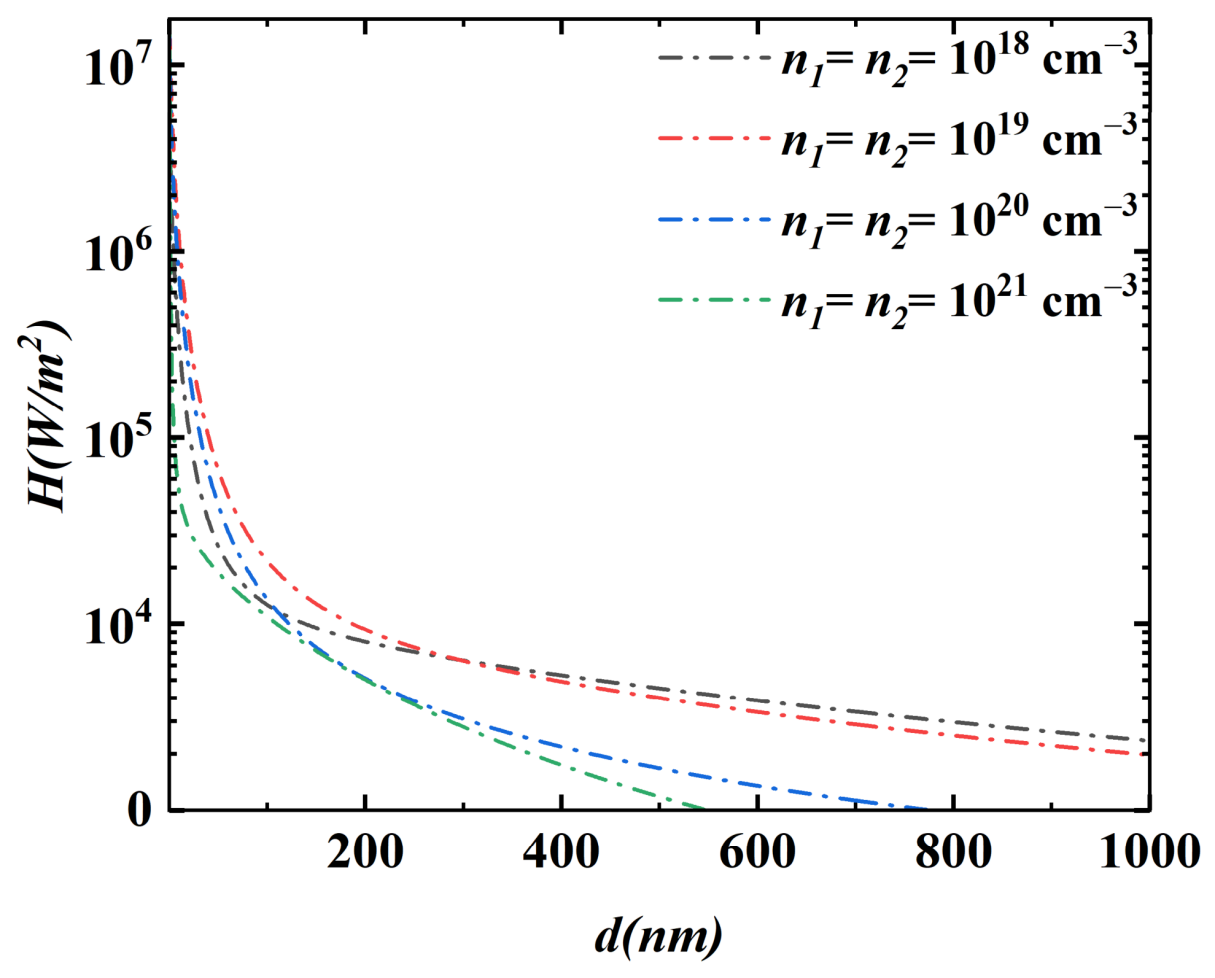
The figure depicts the radiative heat flux of n-type D-Si at different doping concentrations as a function of the vacuum gap distance (d) under the initial conditions of the Drude model parameters.

The dependence of the total radiative heat flux on the vacuum gap distance is presented in Figure 3 for the symmetrically doped cases $n_{1}=n_{2}=n$. A strong near-field enhancement is observed when the gap is reduced to the nanometer scale. At small separations, the heat transfer coefficient exceeds the blackbody limit by several orders of magnitude because of evanescent-wave-induced photon tunneling across the vacuum gap. For all doping concentrations considered, the heat transfer coefficient decreases monotonically with increasing gap distance. This behavior reflects the progressive suppression of evanescent modes as the system moves away from the extreme near-field regime. The enhancement is therefore strongest at very small gap sizes, where the tunneling probability is highest.

A comparison among the different curves further shows that the case $n=10^{19}\;{\mathrm{cm}}^{-3}$ yields the largest total heat transfer at small gap distances, while the $n=10^{18}\;{\mathrm{cm}}^{-3}$ case remains comparable over a relatively broad gap range. In contrast, the $10^{20}$ and $10^{21}\;{\mathrm{cm}}^{-3}$ cases exhibit weaker transfer over most of the range shown. These results indicate that although reducing the vacuum gap is the dominant factor for enhancing near-field radiation, the doping concentration provides an additional and important degree of control over the heat-transfer level.

### 3.3. Spectral Heat Transfer Under Asymmetric Doping

**Figure 4:**
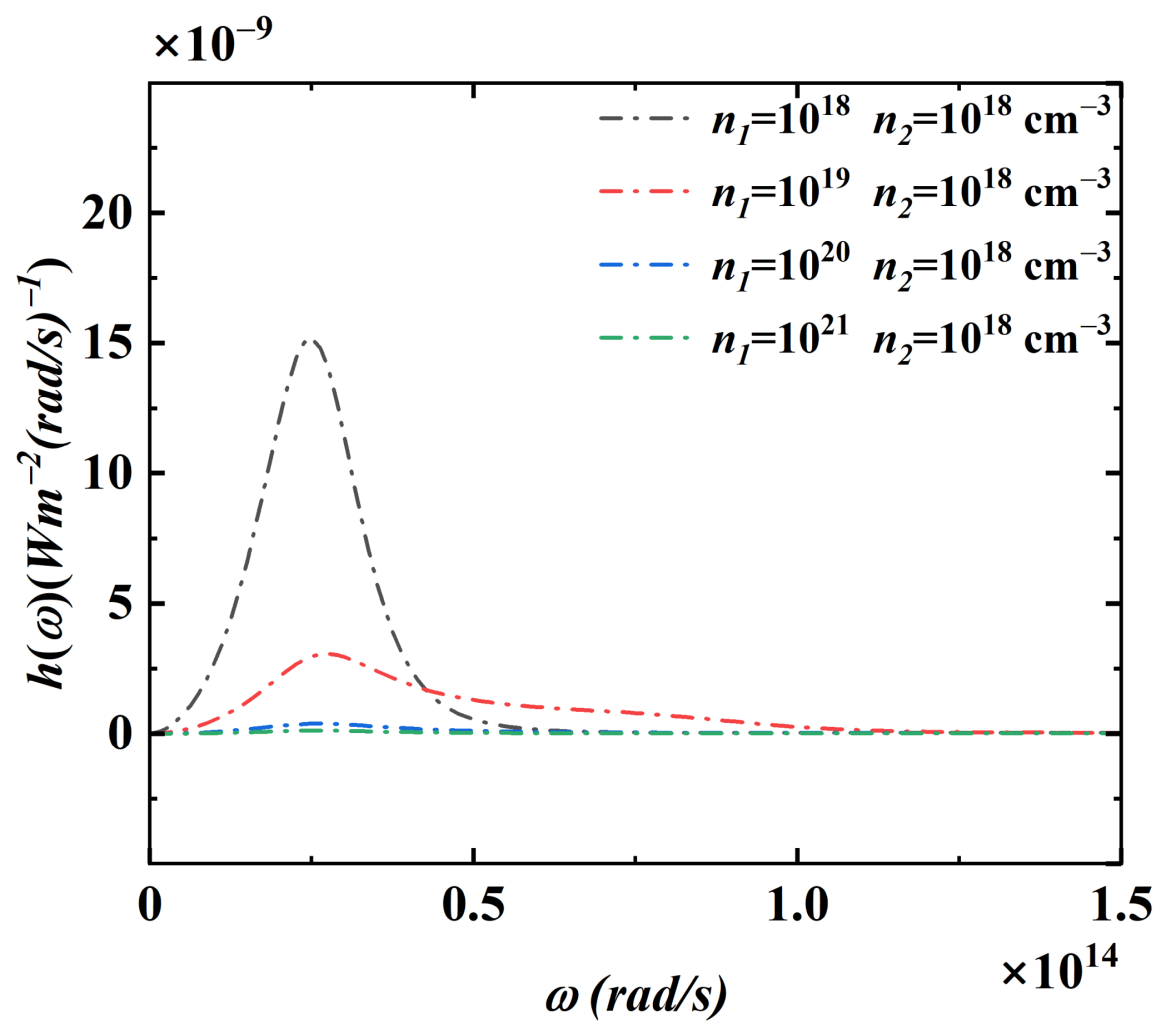
Keeping the doping concentration $n_{2}$ of the lower plate unchanged, the spectral thermal conductance of the upper plate with different doping concentrations $n_{1}$ changes with frequency.

We next examine the influence of asymmetric doping on the spectral heat transfer. In this case, the lower-plate concentration is fixed at $n_{2}=10^{18}\;{\mathrm{cm}}^{-3}$, while the upper-plate concentration $n_{1}$ is varied. The resulting spectral heat flux is shown in Figure 4. It is evident that the overall spectral heat transfer decreases markedly as the upper-plate doping concentration increases from $10^{18}$ to $10^{20}\;{\mathrm{cm}}^{-3}$. The largest spectral peak is obtained for $n_{1}=10^{18}\;{\mathrm{cm}}^{-3}$, where a pronounced low-frequency resonance appears near $3\times 10^{13}\;\mathrm{rad}/s$. When $n_{1}=10^{19}\;{\mathrm{cm}}^{-3}$, the peak intensity drops substantially, but the resonance becomes broader and extends over a wider frequency interval. As $n_{1}$ is further increased to $10^{20}$ and $10^{21}\;{\mathrm{cm}}^{-3}$, the spectral heat transfer is strongly suppressed over nearly the entire frequency range shown.

These results show that, in the asymmetric configuration, increasing the doping concentration of only one plate does not strengthen the radiative coupling. Instead, it weakens the spectral heat flux while partially broadening the resonance at intermediate doping. This behavior can be attributed to the doping-induced change in the dielectric response of the upper plate, which modifies the interfacial optical matching and the effectiveness of the coupled surface-wave channel.

### 3.4. Energy Transmission Coefficient Under Asymmetric Doping

**Figure 5:**
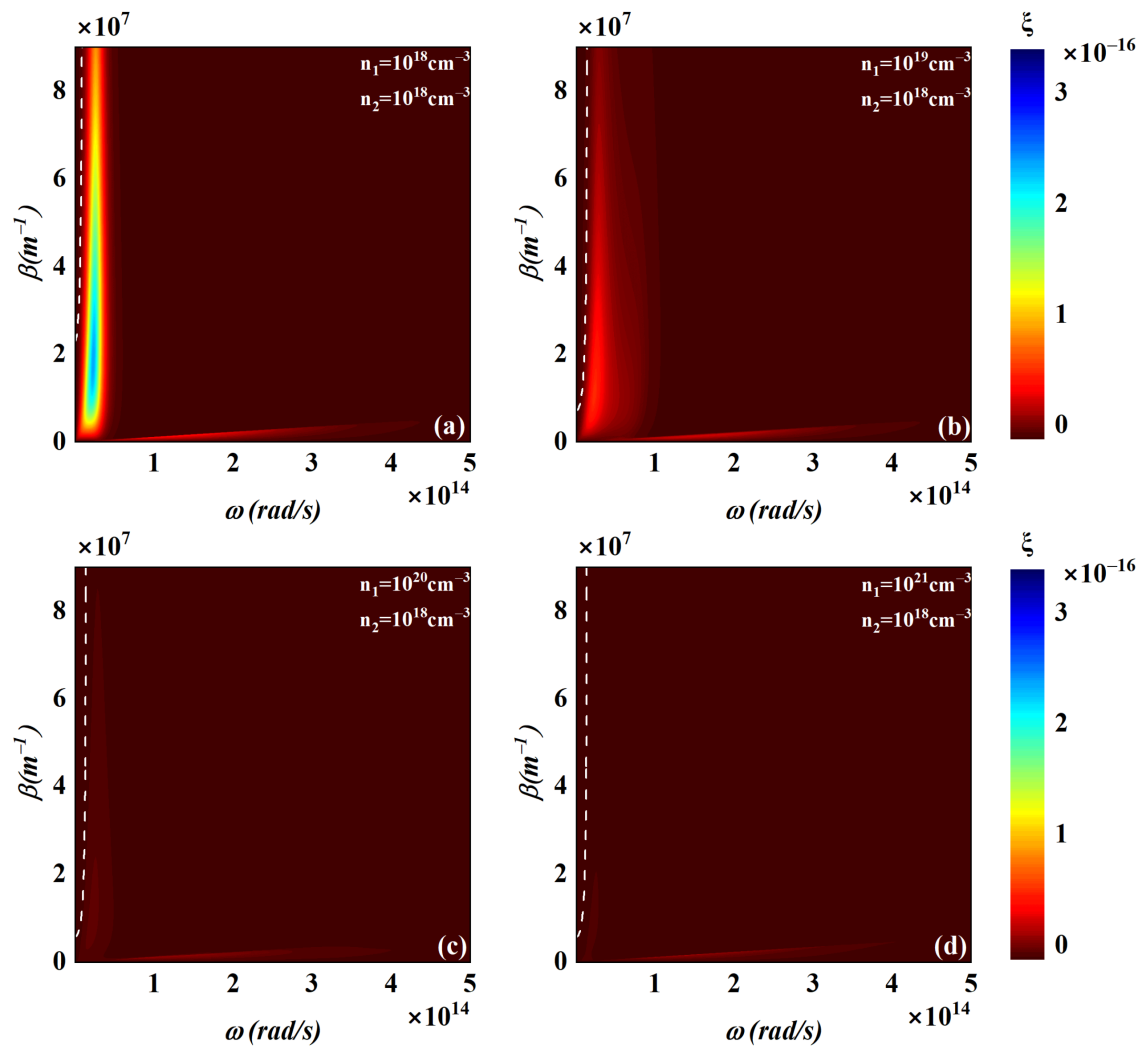
Energy transmission coefficient in the (ω,β) plane for asymmetric doping configurations with the bottom doping concentration fixed at $n_{2}=10^{18}\;{\mathrm{cm}}^{-3}$ and the top doping concentration $n_{1}$ set to $10^{18}$, $10^{19}$, $10^{20}$, and $10^{21}\;{\mathrm{cm}}^{-3}$ (denoted as panels (**a**), (**b**), (**c**), and (**d**), respectively), along with the corresponding SPP dispersion curves (dashed lines).

To elucidate the origin of the spectral evolution in Figure 4, the energy transmission coefficient in the (ω,β) plane is plotted in Figure 5. Here, the SPP dispersion curve is obtained by solving the following dispersion relation: $1-r_{1,p}r_{2,p}e^{2ik_{z}d}=0$, which is derived from the pole condition of the energy transmission coefficient. Here, $r_{1,p}$ and $r_{2,p}$ denote the Fresnel reflection coefficients at the vacuum-upper-plate and vacuum-lower-plate interfaces, respectively; $k_{z}$ is the vacuum wavevector; and d is the separation distance between the two plates [25]. This dispersion curve characterizes the relationship between the angular frequency and the parallel wavevector for the coupled surface electromagnetic modes, thus revealing their propagation and localization properties, as well as their ability to facilitate photon tunneling and near-field radiative heat transfer. The lower plate remains fixed at $n_{2}=10^{18}\;{\mathrm{cm}}^{-3}$, while the upper-plate concentration $n_{1}$ is varied.

As shown in Figure 5, the dominant bright region associated with strong transmission remains in the low-frequency range and exhibits only a relatively small horizontal shift as $n_{1}$ changes. This is because the dispersion curve exhibits no significant horizontal shift. This explains why the peak position in Figure 4 varies only weakly in the asymmetric-doping case. However, the intensity and shape of the bright region change significantly. For $n_{1}=10^{18}\;{\mathrm{cm}}^{-3}$, the transmission map displays the strongest intensity and a narrow bright channel extending to high beta, which corresponds to the largest spectral peak in Figure 4. For $n_{1}=10^{19}\;{\mathrm{cm}}^{-3}$, the bright region becomes broader, although its peak intensity decreases. When n_1_ is further increased to $10^{20}$ and $10^{21}\;{\mathrm{cm}}^{-3}$, the high-β transmission channel is strongly weakened, and the overall transmission becomes much smaller.

Therefore, the reduction in spectral heat transfer with increasing $n_{1}$ is caused mainly by the weakening of the evanescent transmission channel rather than by a large resonance-frequency shift. The comparison between Figure 4 and Figure 5 shows that the asymmetric-doping case is governed primarily by the attenuation and redistribution of the transmission intensity in (ω,β) space.

### 3.5. Spectral Heat Transfer Under Symmetric Doping

**Figure 6:**
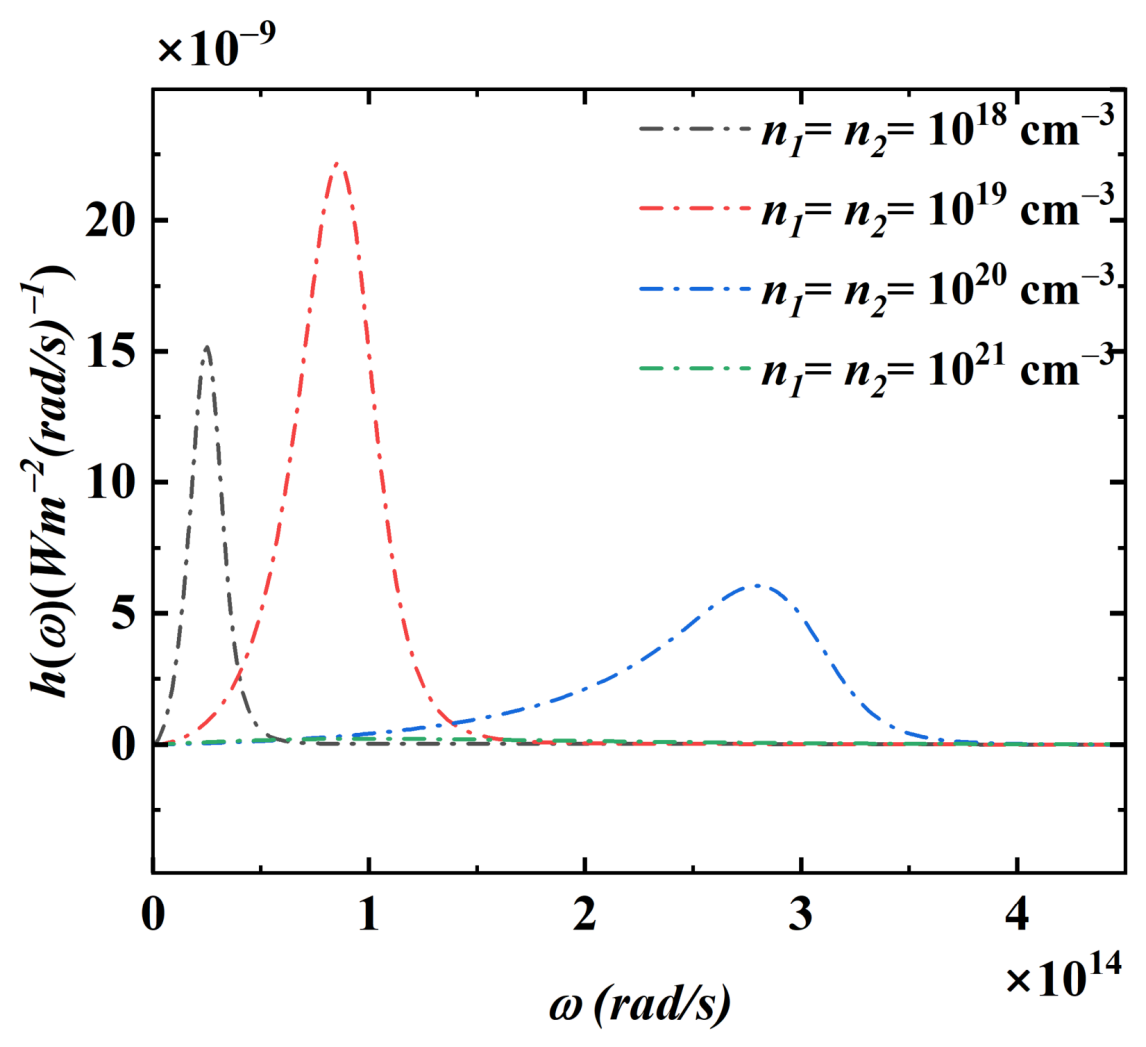
Spectral thermal conductance as a function of frequency for symmetric doping configurations with $n_{1}=n_{2}=n$, where n varies from 10^18^ to 10^21^ cm^−3^.

We now consider the symmetrically tuned case $n_{1}=n_{2}=n$, in which both plates have the same doping concentration. The corresponding spectral heat flux is shown in Figure 6. Compared with the asymmetric case, the symmetric configuration exhibits a much stronger and more structured spectral modulation. As the doping concentration increases from $10^{18}$ to $10^{20}\;{\mathrm{cm}}^{-3}$, the resonance peak shifts clearly toward higher frequencies, indicating a pronounced blueshift of the dominant transfer channel. Specifically, the peak is located near $2\times 10^{13}\mathrm{rad}/s$ for $n=10^{18}\;{\mathrm{cm}}^{-3}$, near $9\times 10^{13}\mathrm{rad}/s$ for $n=10^{19}\;{\mathrm{cm}}^{-3}$, and around $(2.7-3.0)\times 10^{14}\mathrm{rad}/s$ or $n=10^{20}\;{\mathrm{cm}}^{-3}$. At the extreme doping level $n=10^{21}\;{\mathrm{cm}}^{-3}$, however, the resonance is strongly suppressed, and no comparable high-intensity peak is observed. Among all cases, the highest spectral peak is obtained at $n=10^{19}\;{\mathrm{cm}}^{-3}$, while the $n=10^{18}\;{\mathrm{cm}}^{-3}$ case gives the second-largest peak at a much lower frequency. The $n=10^{20}\;{\mathrm{cm}}^{-3}$ curve exhibits a broader but weaker high-frequency resonance, whereas the $n=10^{21}\;{\mathrm{cm}}^{-3}$ curve remains close to zero over almost the entire frequency range. Thus, increasing the doping concentration does not monotonically enhance the spectral heat flux. Instead, the spectral response is determined by the interplay among resonance frequency, coupling strength, and material loss.

The stronger modulation in the symmetric case can be understood by noting that both interfaces can support similar SPP modes. Their coupling across the vacuum gap opens two resonant channels for photon tunneling and therefore yields stronger near-field transfer than in the single-plate tuning case.

### 3.6. Energy Transmission Coefficient Under Symmetric Doping

**Figure 7:**
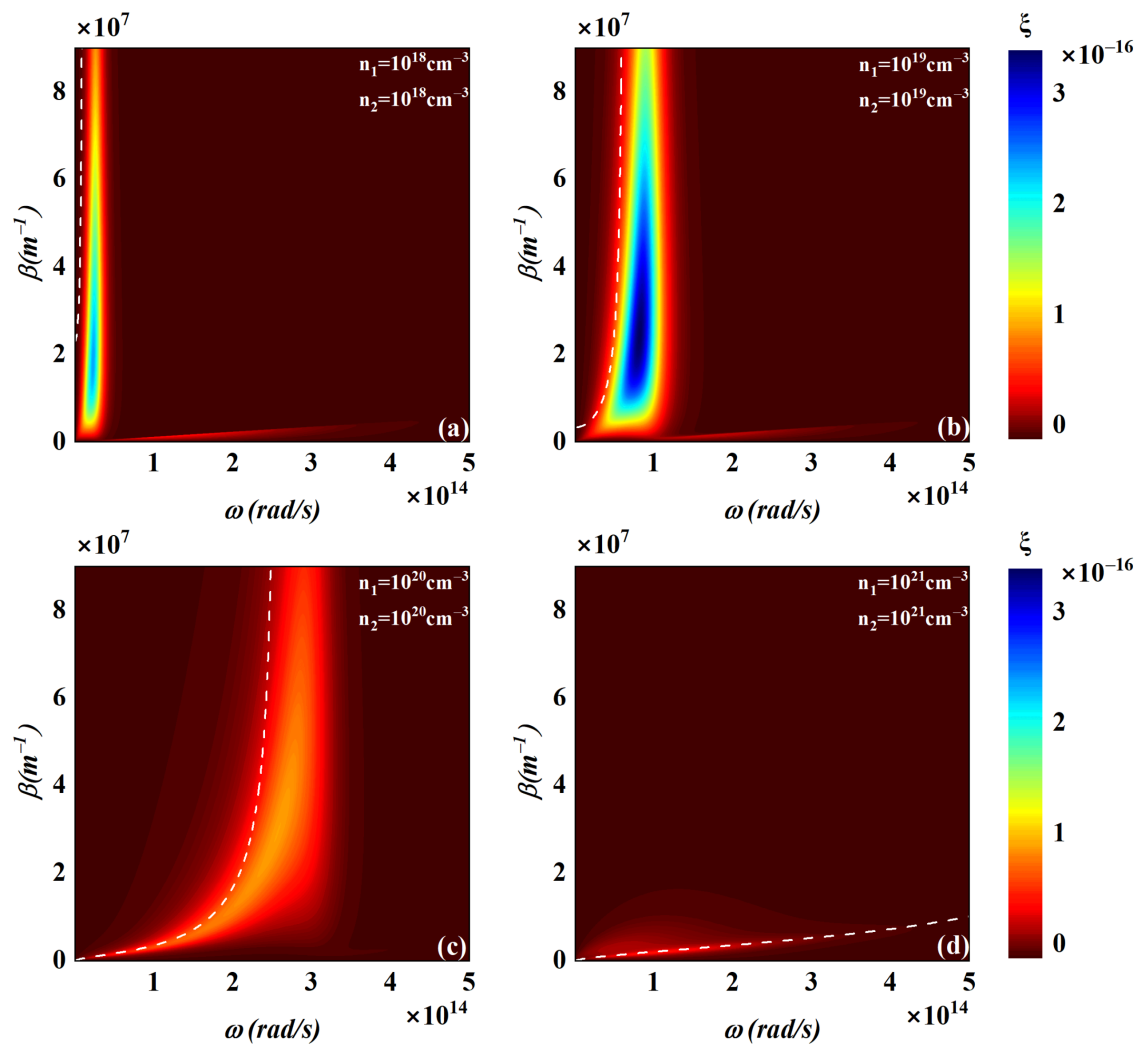
Energy transmission coefficient in the (ω,β) plane for symmetric doping configurations with $n_{1}=n_{2}=n$, where $n=10^{18}$, $10^{19}$, $10^{20}$, and $10^{21}\;{\mathrm{cm}}^{-3}$ (denoted as panels (**a**), (**b**), (**c**), and (**d**), respectively), along with the corresponding SPP dispersion curves (dashed lines).

The underlying mechanism of the symmetric-doping behavior is further illustrated in Figure 7, which shows the energy transmission coefficient in the (ω,β) plane for $n_{1}=n_{2}=n$ with the associated dispersion curves. For $n=10^{18}\;{\mathrm{cm}}^{-3}$, the transmission is concentrated in a narrow low-frequency, high-β band, which is characteristic of a strongly confined evanescent channel. In conjunction with the SPP dispersion curve, when the doping concentration increases to $10^{19}\;{\mathrm{cm}}^{-3}$, the bright region becomes more intense and shifts to higher frequency, consistent with the largest spectral peak observed in Figure 6. At $n=10^{20}\;{\mathrm{cm}}^{-3}$, the high-transmission region undergoes a pronounced blueshift and becomes substantially broader in both frequency and wavevector space, which explains the broadened high-frequency resonance in the spectral heat flux. In contrast, for $n=10^{21}\;{\mathrm{cm}}^{-3}$, the strong high-β transmission channel largely disappears, leaving only a much weaker low-β contribution; correspondingly, the spectral heat transfer is strongly suppressed.

These transmission maps confirm that the near-field coupling between the two plates is highly sensitive to symmetric doping. The transition from a narrow low-frequency channel at $10^{18}\;{\mathrm{cm}}^{-3}$ to a stronger and better-matched channel at $10^{19}\;{\mathrm{cm}}^{-3}$, followed by a broadened but weaker high-frequency channel at $10^{20}\;{\mathrm{cm}}^{-3}$, accounts for the non-monotonic spectral behavior in Figure 6. A comparison between Figure 4 and Figure 6 further shows that the maximum spectral heat transfer is achieved in the symmetrically doped configuration with $n_{1}=n_{2}=10^{19}\;{\mathrm{cm}}^{-3}$. This optimum can be interpreted in terms of the impedance-matching condition for SPP excitation. Within the Drude model, when the real part of the dielectric function approaches −1, the TM reflection factor becomes resonant, and photon tunneling is strongly enhanced. At $n=10^{19}\;{\mathrm{cm}}^{-3}$, the dielectric response provides a favorable balance between resonance matching and damping, resulting in the largest transmission intensity and the highest spectral heat-transfer peak. In all cases considered here, the dominant contribution arises from TM-polarized evanescent modes associated with SPP coupling, whereas the TE contribution is negligible. This is consistent with previous theoretical studies on doped silicon systems, in which the TE-polarized contribution has been quantitatively shown to be several orders of magnitude smaller than the TM counterpart [23,25].

**Figure 8:**
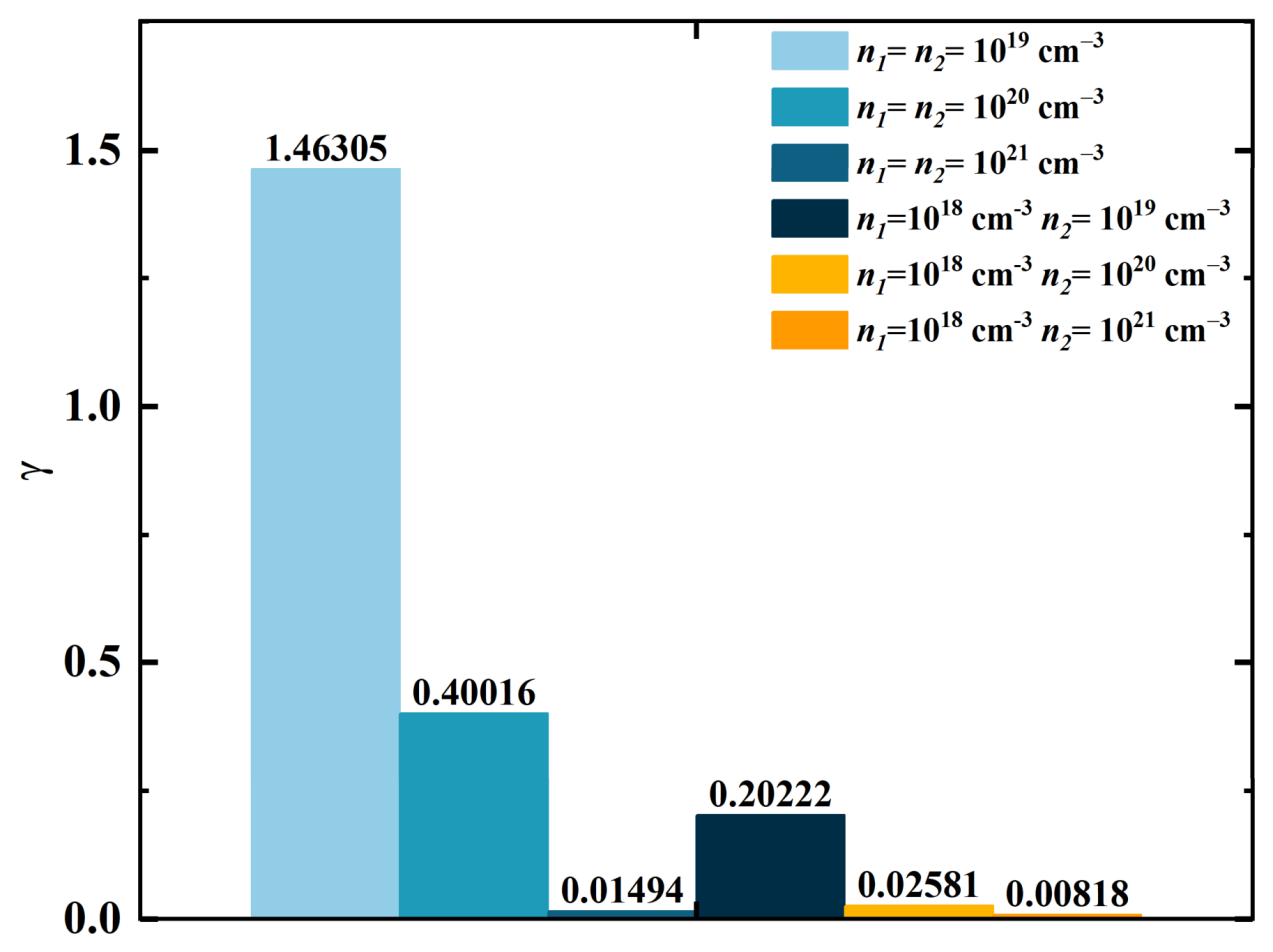
The ratio of the maximum spectral thermal conductance (γ) for six representative doping combinations to that for the symmetric doping configuration $\left(n_{1}=n_{2}=10^{18}\;{\mathrm{cm}}^{-3}\right)$.

To quantitatively compare the heat transfer performance across different doping configurations, we define the ratio of the maximum spectral thermal conductance γ as the peak spectral thermal conductance of a given configuration normalized by that of the symmetrically doped case at $n_{1}=n_{2}=10^{18}\;{\mathrm{cm}}^{-3}$. Figure 8 presents the values of γ for six representative doping combinations. It is evident that, as the doping concentration increases, the maximum spectral thermal conductance exhibits a non-monotonic behavior. Among all cases, the symmetric doping at $n_{1}=n_{2}=10^{19}\;{\mathrm{cm}}^{-3}$ yields the highest ratio of γ=1.46305, confirming that this configuration provides the strongest near-field heat transfer. For the same symmetric concentration, the ratio drops to 0.40016 at $10^{20}\;{\mathrm{cm}}^{-3}$ and further to 0.01494 at $10^{21}\;{\mathrm{cm}}^{-3}$, indicating that excessive doping actually suppresses the spectral heat flux due to increased material loss and detuned SPP resonance. In the asymmetric cases with $n_{2}=10^{18}\;{\mathrm{cm}}^{-3}$, the ratios are significantly lower: γ=0.20222 for $n_{1}=10^{19}\;{\mathrm{cm}}^{-3}$, 0.02581 for $n_{1}=10^{20}\;{\mathrm{cm}}^{-3}$, and 0.00818 for $n_{1}=10^{21}\;{\mathrm{cm}}^{-3}$. These quantitative results clearly demonstrate that symmetric doping at the optimal concentration of $n_{1}=10^{19}\;{\mathrm{cm}}^{-3}$ is essential for maximizing near-field radiative transfer, and that any deviation from this condition, either through asymmetric doping or excessive symmetric doping, leads to a measurable degradation in performance. The spectral peak positions in Figure 6 coincide with the frequencies at which the SPP dispersion curves in Figure 7 cross the regions of maximum transmission intensity. For $n=10^{19}\;{\mathrm{cm}}^{-3}$, the SPP resonance frequency falls within the thermal radiation window (weighted by the Planck function), yielding the strongest spectral peak. For $n=10^{20}\;{\mathrm{cm}}^{-3}$, the resonance shifts to higher frequencies where the thermal excitation is weaker, while for $n=10^{18}\;{\mathrm{cm}}^{-3}$, the resonance lies at lower frequencies where the SPP coupling is less efficient.

## 4. Conclusions

In summary, near-field radiative heat transfer between two heavily phosphorus-doped silicon plates has been studied theoretically using fluctuational electrodynamics combined with a doping-dependent Drude model. The results show that nanoscale vacuum gaps produce a heat-transfer coefficient several orders of magnitude larger than the blackbody limit because of evanescent-wave tunneling. The dielectric response of heavily doped n-type silicon is strongly affected by doping concentration. As the angular frequency increases, the real part of the permittivity evolves from large negative values toward less negative or positive values, while the imaginary part decreases monotonically. These doping-dependent optical properties strongly influence both the spectral and the total radiative transfer. For asymmetric doping, increasing the upper-plate concentration while keeping the lower plate fixed at $10^{18}\;{\mathrm{cm}}^{-3}$ causes a substantial reduction in the spectral heat flux, although the resonance becomes broader at intermediate doping. For symmetric doping, the spectral peak shifts toward higher frequencies as the doping concentration increases from $10^{18}\;{\mathrm{cm}}^{-3}$ to $10^{20}\;{\mathrm{cm}}^{-3}$, and the strongest transfer is obtained at $n_{1}=n_{2}=10^{19}\;{\mathrm{cm}}^{-3}$. The quantitative comparison of the maximum spectral thermal conductance ratio further confirms that the optimal heat transfer is achieved exclusively under the symmetrically doped condition $n_{1}=n_{2}=10^{19}\;{\mathrm{cm}}^{-3}$, with a ratio of 1.46305 relative to the reference case. At this concentration, the dielectric response best satisfies the impedance-matching condition for SPP-mediated photon tunneling, yielding the largest transmission intensity and the maximum heat-transfer coefficient. These findings provide clear physical insight into the doping-controlled near-field thermal radiation in CMOS-compatible doped-silicon systems, and establish a useful theoretical foundation for chip-scale thermal management, hotspot mitigation, and adaptive radiative cooling in advanced integrated circuits. Future work may further explore asymmetric doping gradients and many-body near-field thermal transport in related doped-silicon configurations for high-power-density and three-dimensional chip applications. In particular, practical tuning mechanisms and device-level analyses remain important directions for bridging the gap between fundamental theory and realistic thermal management applications.

## Figures and Tables

**Figure 2:**
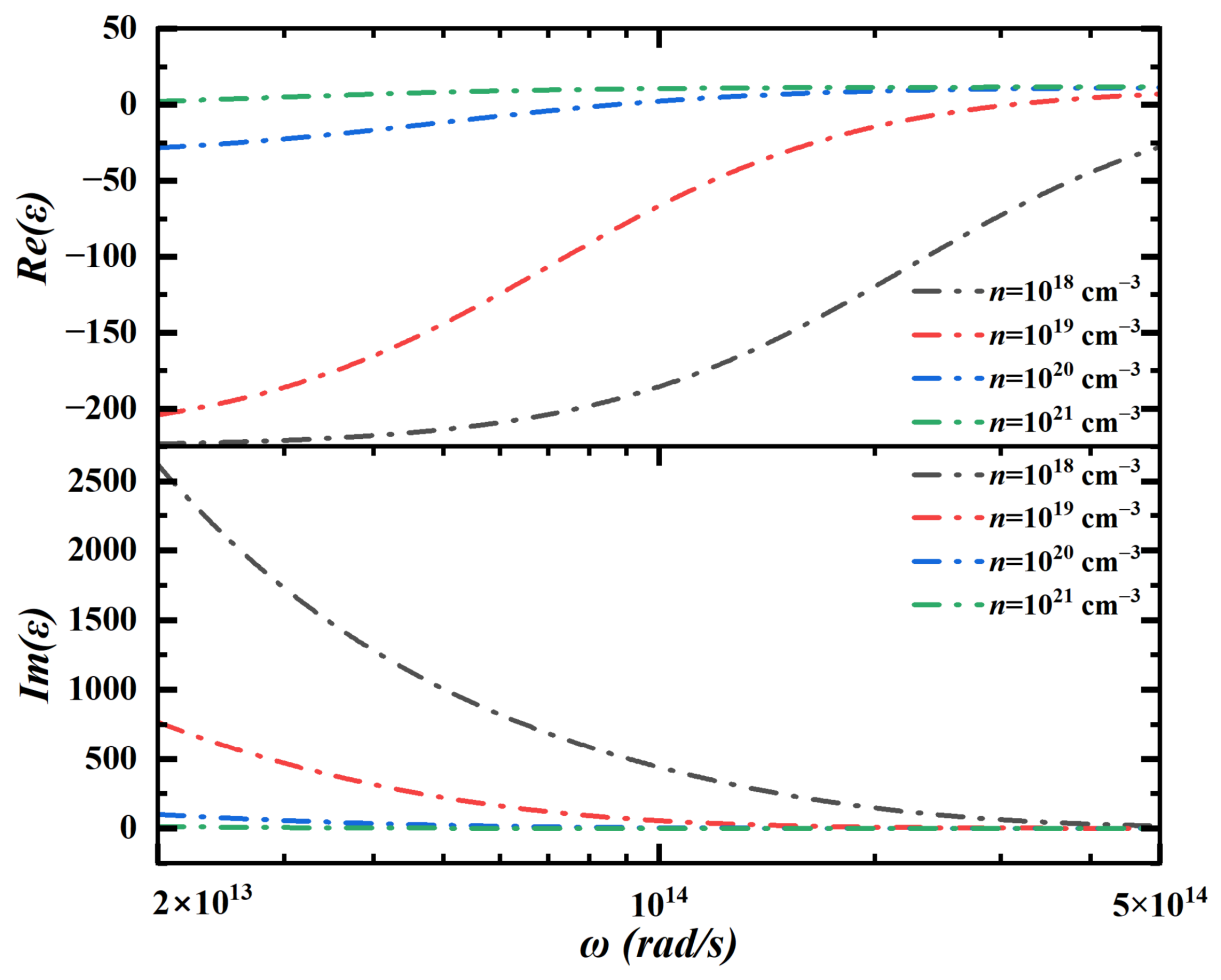
Variation in the real and imaginary parts of the n-type D-Si dielectric constant with angular frequency at different doping concentrations.

**Table 1:** Carrier concentration, mobility, and relaxation time of n-type doped silicon at 300 K and 400 K for various doping levels.

Sample	Doping Concentration	Carrier Concentration (400 K) $\left(10^{17}\right)$	Mobility	Relaxation Time $\left(10^{-13}\right)$	Carrier Concentration (300 K) $\left(10^{17}\right)$	Mobility	Relaxation Time $\left(10^{-13}\right)$
1	$10^{18}$	9.536	442.481	0.356	9.216	287.400	0.441
2	$10^{19}$	99.077	176.968	0.199	99.501	114.944	0.176
3	$10^{20}$	999.998	112.741	0.141	1000.000	73.227	0.112
4	$10^{21}$	10000.000	31.055	0.045	10000.000	20.171	0.031

**Table 2:** Comparison of the present work with previous theoretical investigations on near-field radiative heat transfer involving doped silicon.

Aspect	Zhang [26]	Lim [25]	Present Work
Material system	Various materials	P-doped Si and graphene	P-doped Si only
Symmetric vs. Asymmetric doping	Not distinguished	Not distinguished	Systematic comparison
Quantitative metric for heat transfer performance	No	No	Yes
CMOS compatibility emphasis	No	No	Yes
Quantitative comparison of the best doping concentration	-	-	Yes

## Data Availability

The data presented in this study are available on request from the corresponding author.

## Abbreviations

The following abbreviations are used in this manuscript:

d	vacuum gap distance, nm
$T_{1}$	temperature of the upper plate, K
$T_{2}$	temperature of the lower plate, K
H	$\mathrm{radiative}\;\mathrm{heat}\;\mathrm{flux},\;{W/m}^{2}$
h	$\mathrm{spectral}\;\mathrm{thermal}\;\mathrm{conductance},\;{\mathrm{nJm}}^{-2}K^{-1}$
Θ	Planck oscillator’s average energy, J
ω	angular frequency, rad/s
ξ	energy transmission coefficient
β	$\mathrm{in}\text{-}\mathrm{plane}\;\mathrm{wave}\;\mathrm{vector},\;m^{-1}$
$k_{x}$	$\mathrm{in}\text{-}\mathrm{plane}\;\mathrm{wave}\;\mathrm{vector}\;(x\;\mathrm{component}),\;m^{-1}$
$k_{y}$	$\mathrm{in}\text{-}\mathrm{plane}\;\mathrm{wave}\;\mathrm{vector}\;(y\;\mathrm{component}),\;m^{-1}$
ℏ	reduced Planck constant, Js
$k_{B}$	Boltzmann constant, J/K
$r_{i}^{j}$	reflection coefficient
$t_{i}^{j}$	transmission coefficient
ε	dielectric function
$\varepsilon_{\infty }$	high-frequency dielectric constant
$\omega_{p}$	plasma frequency, rad/s
τ	scattering time, s
$N_{e}$	$\mathrm{electron}\;\mathrm{concentration},\;{\mathrm{cm}}^{-3}$
e	electron charge, C
$m^{*}$	electron effective mass, kg
ζ	degree of ionization
$\varepsilon_{0}$	vacuum permittivity
$\tau_{e-d}$	electron-defect scattering time, s
$\tau_{e-l}$	electron-lattice scattering time, s
μ	$\mathrm{electron}\;\mathrm{mobility},\;{\mathrm{cm}}^{2}/\mathrm{Vs}$
$m_{e}$	free-electron mass, kg
c	speed of light in vacuum, m/s
$n_{1}$	$\mathrm{doping}\;\mathrm{concentration}\;\mathrm{of}\;\mathrm{the}\;\mathrm{upper}\;\mathrm{plate},\;{\mathrm{cm}}^{-3}$
$n_{2}$	$\mathrm{doping}\;\mathrm{concentration}\;\mathrm{of}\;\mathrm{the}\;\mathrm{lower}\;\mathrm{plate},\;{\mathrm{cm}}^{-3}$
γ	ratio of maximum spectral thermal conductance
